# The Antidepressant and Cognitive Improvement Activities of the Traditional Chinese Herb* Cistanche*

**DOI:** 10.1155/2017/3925903

**Published:** 2017-06-28

**Authors:** Dongfang Wang, Haizhen Wang, Li Gu

**Affiliations:** ^1^State Key Laboratory of Biocontrol, Institute of Aquatic Economic Animals and Guangdong Province Key Laboratory for Aquatic Economic Animals, School of Life Sciences, Sun Yat-sen University, Guangzhou 510275, China; ^2^Food and Health Engineering Research Center of State Education Ministry, Sun Yat-sen University, Guangzhou 510275, China

## Abstract

More than ten percent of people suffer from at least one episode of depression and related mental disorders in a lifetime, and depression and related mental disorders are one of the world's greatest public health problems. A multiple system theory holds that dysregulation of the multiple systems underlies the pathogenesis of depression and related mental disorders, and new therapies based on the multiple system dysregulation theory are urgently needed. In this study, the antidepressant effect of decoction from herb* Cistanche deserticola* Y.C.Ma and* Cistanche tubulosa* was examined. Herb* Cistanche* decoction reduced the immobility period significantly in the mouse tail suspension test. Mice treated with herb decoction showed an improved ability of spatial learning and memory in the Morris water maze test. Groups treated herb decoction displayed a downregulated monoamine oxidase (MAO) activity; the dopamine (DA) concentration in the brain was upregulated, indicating herb* Cistanche* decoction improved the nerve excitability; the serum concentration of corticosterone (CORT) was downregulated, showing that mice benefited from a reduced stress level. Hence, the antidepressant efficacy and mechanism of traditional Chinese herb* Cistanche* were explored in this study. Herb* Cistanche* showed a potential to be developed as a complementary and alternative therapy for depression.

## 1. Introduction

Depression and related mood disorders, with an estimate of 12~17% of the population experiencing at least one episode in a lifetime, are among the leading causes of mental disabilities and considered as one of the worldwide major public health problems [1]. Of particular relevance, a significant proportion of patients with depression suffer from memory loss, concentration problems, decreased energy or fatigue, loss of interest or pleasure in hobbies and activities, and difficulty in sleeping, early-morning awakening, or oversleeping. Although the neural substrates for abnormality in depression and related syndromes are unclear, it is generally accepted that the impairment of neuroplasticity and cellular resilience gives rise to the pathophysiology of depression and successful treatment may depend on the intervention of the neurotransmitter level [2]. The approach to depression medication is basically based on the classic monoamine system hypothesis, which claims that dysregulation of monoamines especially serotonin (5-HT) and norepinephrine (NE) contributes to the depression pathogenesis, and intervention of the neurotransmitter system with dopamine agonists and dopamine-noradrenergic modulators would alleviate the symptoms of major depression disorder [3]. Although widely prescribed, the antidepressants curative efficacy is questionable and the side-effect cannot be ignored because a significant proportion of the patients do not respond to the monoamine system manipulation [4]. Recently, novel theories of depression pathogenesis indicate that multiple systems, including inflammatory pathways, the oxidative stress pathway, the hypothalamic-pituitary-adrenal (HPA) axis, the metabolic and bioenergetics system, neurotrophic pathways, the glutamate system, the opioid system, and the cholinergic system, are closely associated with the depression occurrence [3]. Evidences show that multiple target intervention produced better efficacy than one single target intervention [5]. New therapies based on the multiple system dysregulation theory are urgently needed.

Herbal products reportedly consumed more than half of US population [6]; interest and demands for herb or herbal products as a supplemental way of treating depression are growing all over the world. Multiple medical plants are found to demonstrate depression-relieving beneficial effect. Herbs that are reported to possess the effect of suppressing depression and improving cognitive function include sea buckthorn [7],* Ginkgo biloba *[8],* Piper nigrum *[9],* Hypericum perforatum *[10],* Griffonia simplicifolia *[11]. A combination administration of ferulic acid and piperine displayed a synergistic antidepressant-like effect in mice by manipulating the monoaminergic system [7]. Albiflorin was characterized by a high selective affinity to the neurotransmitter receptors and transporters and significantly increased extracellular concentrations of 5-HT and NE in the hypothalamus of freely moving rats [12]. A medical herb* Melissa officinalis* L. relieved the depressive-behavior of mice by exerting the influence on the the serotonergic neurotransmitter level [13]. These herbs or decoctions, developed as the alternative remedies for depression, help alleviate the symptoms of depression and provide a new source for the antidepressant drug developing. Herb* Cistanche* species, distributed in arid lands and deserts northern part of China, are traditional Chinese medicine herbs and widely used for treating various diseases for more than 1,000 years in China. The multiple efficacy of herb* Cistanche* decoction ranges between the aphrodisiac [14], immune-promoting [15], antioxidative [16], and hepatoprotective [17] properties.* C. deserticola *decoctions induce the testicular steroidogenic enzymes [18], promote penis erectile response, and shorten the latency period of erectile reaction in castrated rats [19]. Herb* Cistanche* has been reported to inhibit the activities of dopaminergic neurons by regulating the inhibitory apoptosis genes and neurotrophic factors [20].* C. tubulosa *decoctions amended the cognitive dysfunction caused by A*β* 1-42 via blocking amyloid deposition and revived the cholinergic and hippocampal dopaminergic neuronal function [21]. The neuroprotective property of herbal* Cistanche* implies the therapeutic potential in the cognitive-related diseases. Depression is a common mixed mode of emotion reactions with forgetfulness and cognitive decline as the common symptoms [22]. Herb* Cistanche* was commonly used as a traditional Chinese medicine to treat forgetfulness; moreover, a recent report shows that an early-adult outset administration of the cornmeal supplemented with* C. tubulosa *is beneficial for the longevity promotion and olfactory-associated learning and memory improvement in the nonvertebrate* Drosophila* model [23]. These facts indicate the cognitive improving property of herb* Cistanche*; here in this research, for the first time we characterized the antidepressant property and its relationship to cognitive improvement activities in the mammal model by regulating the monoamine system and HPA axis. Illuminating the antidepressant property and preliminary mechanism of herb* Cistanche* could expand the application spectrum of herb* Cistanche*, which could be developed as an alternative remedy for depression.

## 2. Materials and Methods

### 2.1. Plant Material and Decoction Preparation

*C. deserticola* Y.C.Ma was obtained from the Nei Mongol Autonomous Region, China;* C. tubulosa* was obtained from the Xinjiang Uygur Autonomous Region, China. Before decoction, the herb (*C. deserticola* Y.C.Ma or* C. tubulosa*) was inspected to detect the content of echinacoside and verbascoside using HPLC (high performance liquid chromatography) (Milford, MA, Waters, USA), and the eligible product (according to Pharmacopoeia of the People's Republic of China) was used for the animal experiments. The decoction was acquired based on the method of water vaporing developed by Food and Health Engineering Research Center of State Education Ministry, Sun Yat-sen University. Dried* Cistanche deserticola* Y.C.Ma of 125 g was smashed and sieved by 120-mesh screen and then powder was dissolved in 3 L ultrapure water, heated at 100°C for 2 h, then cooled down to room temperature, and sieved; the supernatant was recycled. The sediment was then dissolved in 2 L ultrapure water and then heated for 1.5 h and cooled down as mentioned above to recycle the supernatant; the sediment was then used for recycling the supernatant. The content of all the supernatant was enriched by rotary vacuum evaporators at 60°C; finally the crude drug concentration was set as 0.5 g/ml and the product was stored at −20°C.

### 2.2. Echinacoside and Verbascoside Assay by HPLC Analysis

HPLC (high performance liquid chromatography) was used to measure the qualitative and quantitative assay of echinacoside and verbascoside; verbascoside (purity > 93%) and echinacoside (purity > 93%) were purchased from the National Institutes for Food and Drug Control (Beijing, China) and utilized as a standard control.

### 2.3. The Animal Care and Experimental Conduct

The subjects were SPF (specific pathogen-free) male Kunming mice weighing 21~25 grams, supplied by Medical Experimental Animal Center of Guangdong Province. The animals were housed in plastic cages (ten per cage); the environment condition was set such as follows: humidity 40~70%, room temperature 21 ± 1°C, and a 12 h : 12 h light-dark period. All animal experiments were approved by the Animal Ethics Committee of Sun Yat-sen University.

### 2.4. Tail Suspension Test

The tail suspension test was done according to the protocol described by Cryan et al. [24] with modest changes. Briefly, on day 21, the mice were suspended by the tail using metallic gallows tethered by nylon catheter, with head at the height 50 mm from the floor. The mice were hung on the hook on adhesive tapes 10~20 mm from the extremity of its tail and were isolated acoustically and visually isolated by clapboards. The body movements of the mouse were recorded and the respiratory movements were ignored. Before the day of experiment, the mice were suspended for 8 min for adaptation.

### 2.5. The Morris Water Maze Test

Water maze test was done according to the description of Vorhees et al. [25–27] using the device developed by Chinese Academy of Medical Sciences. A circular pool (150 cm diameter) filled with water (26 ± 1°C; rim height (distance water surface to wall rim): 10 cm) was used, circular rescue platform (diameter: 11 cm; distance between platform center point and pool wall: 27 cm) was submerged 1–1.5 cm below the water surface, and the testing area was illuminated with indirect lighting (50 ± 10 lux) to avoid reflections. Four start locations were used, N, S, E, and W. For the spatial learning test, animals were trained daily on days 9–11 on the fixed start location. The escape latency period is measured to display the spatial memory and learning ability of the mice. If the mice could not find or climb onto the platform, the mice would be leaded to the platform and stay for 10 s. After taking a rest for 60 s, then next session of training would be started. Data obtained from the last spatial learning test were used as base value for molding. On days 23–28, place navigation test and spatial probe were done. The place navigation test lasted for 6 days and was taken on 1 o'clock p.m., animals were placed into the water with their head facing the pool wall clockwise, and the latency period (within 2 min) was recorded. After the place navigation test was done, the platform was removed. The mice were placed into water with their head facing the pool wall at a random location, and the motion trail and the frequency that mice swam across where the platform was within 2 min were recorded. The motion trail was recorded by multifunction autonomic recorder of mice motion (YLS-1A, Yiyan Technology, Jinan, China).

### 2.6. Detection of the MAO Activity

The MAO activity was measured using MAO detection kit (A034, Nanjing Jiancheng Bioengineering Institute, Nanjing, China) and the detection was done according to the manufacture's protocol. Homogenate from brain tissue was obtained; 500 *μ*l was mixed with 300 *μ*l of Agent 1 and 300 *μ*l of Agent 2, incubated for 3 h at 37°C, and then successively added with 300 *μ*l, of Agent 3, 3 ml of Agent 4, and vortex for 2 min. The reaction mixture was centrifuged 3,000 rpm for 10 min, and the optic density of the supernatant was determined by ultraviolet and visible spectrum spectrophotometer (Lambda 25, PerkinElmer, USA) using 242 nm excitation light.

### 2.7. Enzyme Linked Immunosorbent Assay of Neurotransmitters and ACTH and CORT

Enzyme Linked Immunosorbent Assay (ELISA) detection kit for dopamine (DA), norepinephrine (NE), and 5-hydroxytryptamine (5-HT) was bought from BLUE GENE, Shanghai. ELISA detection kits were bought from Qiyun Biotech, Guangzhou.

The procedures are such as follows: the experiment was conducted at room temperature. Coat the wells of a PVC microtiter plate with the capture antibody at 1–10 *μ*g/mL concentration in carbonate/bicarbonate buffer (pH 9.6). Seal the plate and incubate overnight 4°C; wash the plate with PBS/tween 3 times, and the nonspecific binding sites are blocked by Block Solution; then wash the plate with PBS/tween 3 times. Standards and samples are diluted in Blocking Solution and 100 *μ*l is added in the well of the plate, and the plate is sealed and incubated at 37°C for 1 h. Add 100 *μ*l diluted detection antibody and incubate the plate at 37°C for 1 h; wash the plate with PBS/Tween 3 times. Add 50 *μ*l color development solution and keep the plate away from the light and incubate it at room temperature for 15 min; 50 *μ*l stop buffer is added to stop the reaction. Measure the optical density (OD) for each well with a microplate reader set to 405 nm in 30 min.

### 2.8. Statistical Analysis

All the data were represented as mean ± SEM (standard error of the mean), the significance difference was analyzed by* t*-test or one-way ANOVA followed by Duncan's multiple range test using SPSS 16.0, and differences between groups with *P* < 0.05 were considered statistically significant.

## 3. Results

### 3.1. Determining the Content of Main Bioactive Ingredients in the* C. deserticola* Y.C.Ma and* C. tubulosa* Samples

**Figure 1:**
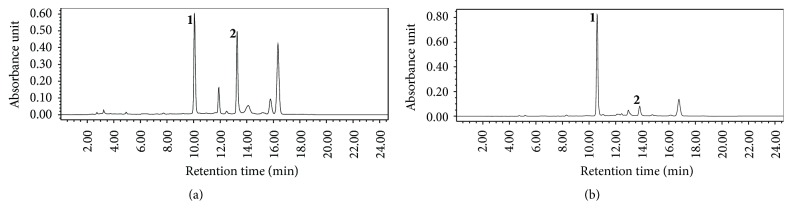
The HPLC chromatographic profile of phenylethanoid glycosides from the* C. deserticola* Y.C.Ma and* C. tubulosa* powder. Peaks 1 and 2 represent echinacoside and verbascoside, respectively.

The phenylethanoid glycosides such as echinacoside and verbascoside are generally considered as the main bioactive ingredients; hence the content of echinacoside and verbascoside in both* C. deserticola* Y.C.Ma and* C. tubulosa* samples was determined by HPLC. The HPLC chromatograms were shown in Figure 1, and the results show that the total content of phenylethanoid glycosides from* C. deserticola* Y.C.Ma was approximately twice that from* C. tubulosa*. Echinacoside and verbascoside weights, respectively, account for 1.27 ± 0.009% and 0.52 ± 0.003% of powder prepared from* C. deserticola* Y.C.Ma and 19.81 ± 0.66% and 2.45 ± 0.12% of powder prepared from* C. tubulosa*. Echinacoside and verbascoside weights, respectively, account for 0.90 ± 0.001% and 0.20 ± 0.001% of* C. deserticola* Y.C.Ma decoction and 5.90 ± 0.12% and 0.54 ± 0.05% of powder prepared from* C. tubulosa* (Table 1).

### 3.2. Decoction of* C. deserticola* Y.C.Ma and* C. tubulosa* Alleviated the Stress and Depression Level

**Figure 2:**
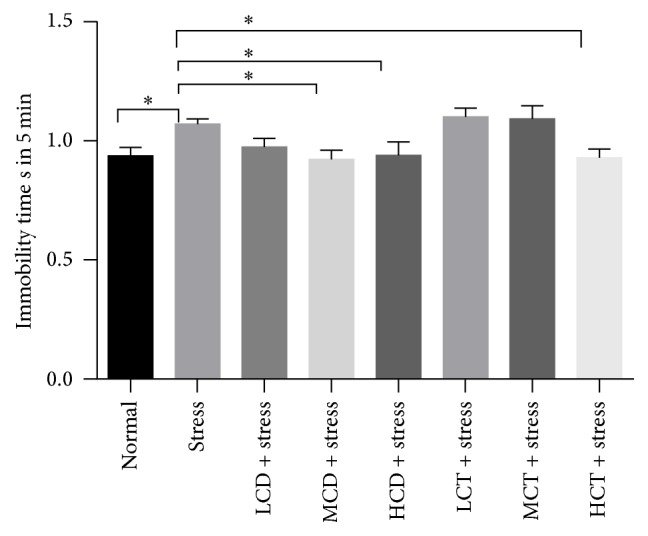
Effect of* C. deserticola* Y.C.Ma and* C. tubulosa* decoctions on immobility period of tail suspension mice in 5 min. After adaptation, the experimental groups were administered with herb* Cistanche* decoction by gavage for 21 days and then suspended by tail and the movement was monitored in 5 min after suspension; each group was comprised of 10 mice. L, M, and H, respectively, represent low, medium, and high; CD and CT, respectively, represent* C. deserticola* Y.C.Ma and* C. tubulosa* decoction. All the data were represented as mean ± SEM; *P* < 0.05 was considered as significantly different and was marked as “*∗*”.

Tail suspension test (TST) is a quick and classic method to assess the antidepressant effect of drugs in mice [24]; in this research, the immobile status, caused by the short-term and inescapable stress of being suspended by the tail, was used as a trait to reflect the depression level of mice. We checked whether decoction of* C. deserticola* Y.C.Ma and* C. tubulosa* could reverse the immobility and promote the occurrence of escape-related behavior. Compared to control groups, the immobility period of the stress group increased by 27.4%; compared to the stress groups, the groups treated with medium, high concentration of* C. deserticola* Y.C.Ma herb decoction showed a statistically significant decrease of immobility period each by 44.1% and 56.1%; the groups treated with medium, high concentration of* C. tubulosa* herb decoction showed a statistically significant decrease of immobility period each by 41.9% and 47.7% (Figure 2). These results indicate that decoction of* C. deserticola* Y.C.Ma and* C. tubulosa *could decrease the stress and depression level of mice, and* C. deserticola* Y.C.Ma decoction exhibited a slightly higher efficacy than* C. tubulosa* decoction.

### 3.3. Decoction of* C. deserticola* Y.C.Ma and* C. tubulosa* Improved the Spatial Learning and Memory of Mice

People (both adolescent and adult) afflicted to depression are prone to suffering learning disability [28]; decoction of* C. deserticola* Y.C.Ma and* C. tubulosa *was proved to alleviate the stress and depression level; then we chose to assess whether decoction of* C. deserticola* Y.C.Ma and* C. tubulosa* could affect the spatial learning and memory by using the Morris water maze model, which is widely applied in assessing the spatial learning and memory function of related brain area [29]. The motion trails are classified into four during the search latency; a straight line trajectory showed the mice pinpointed the rescue platform's position, a tendency trajectory showed that the mice exhibited a basically right orientation, and the edgy type trajectory showed that the mice either were in the early training period or suffered from dementia, trying to locate target place based on instinct; the random searching trajectory showed that mice had low ability of positioning and searched the target place aimlessly. Data were acquired according to the criteria.

**Figure 3:**
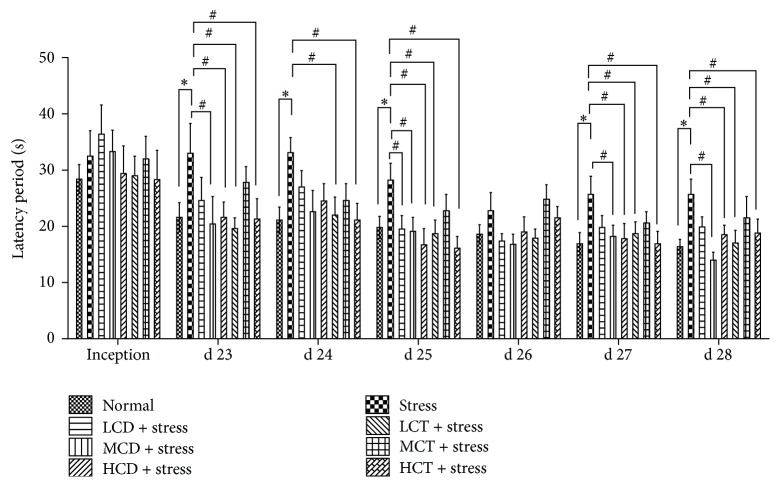
The effect of* C. deserticola* Y.C.Ma and* C. tubulosa* decoctions on the space learning and memory capability of the restraint stressed mice. After adaptation, the experimental groups were administered with herb* Cistanche* decoction by gavage for 21 days and then tested with R. Morris water maze for determination of spatial learning and memory. L, M, and H, respectively, represent low, medium, and high; CD and CT, respectively, represent* C. deserticola* Y.C.Ma and* C. tubulosa* decoction. “#” and “*∗*” each represent *P* < 0.05 and *P* < 0.01.

Effect of* C. deserticola* Y.C.Ma and* C. tubulosa* decoctions on the space learning and memory capability of restraint stressed mice is shown in Figure 3. After tail suspension test, mice showed a significantly prolonged latency period. Compared to the stress groups, the mice treated moderate, high dosage* C. deserticola* Y.C.Ma and low, high dosage* C. tubulosa* decoction showed a shortened latency period; the frequency that mice swam the hidden platform was upregulated significantly in the groups treated with moderate, high dosage* C. deserticola *Y.C.Ma decoction. These results indicate that the stressed mice benefited from the administration of herb* Cistanche* decoction and displayed an improvement of the spatial learning and memory.

### 3.4. Decoction of* C. deserticola* Y.C.Ma and* C. tubulosa *Downregulated the Monoamine Oxidase Activity

**Figure 4:**
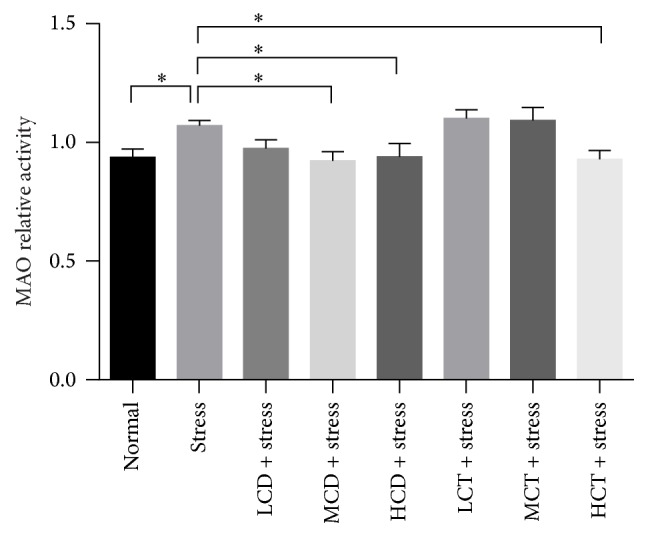
Effect of* C. deserticola* Y.C.Ma and* C. tubulosa* decoctions on brain monoamine oxidase (MAO) activity of tail suspension stressed mice. After adaptation, the experimental groups were administered with herb* Cistanche* decoction by gavage for 21 days and then suspended by tail for 5 min, each group was comprised of 10 mice, and the mice were sacrificed to harvest the brain tissues and used for MAO activity measurement. L, M, and H, respectively, represent low, medium, and high; CD and CT, respectively, represent* C. deserticola* Y.C.Ma and* C. tubulosa* decoction. *n* = 10; all the data were represented as mean ± SEM; *P* < 0.05 was considered as significantly different and was marked as “*∗*”.

MAO catalyzes the oxidative deamination of bioamines such as tyramine, catecholamine, and 5-hydroxytryptamine (5-HT) in the brain and peripheral tissues; inhibition of MAO activity results in the antidepressant efficacy [30]. We sought to explain the antidepressant effect by* C. deserticola* Y.C.Ma and* C. tubulosa*; then the MAO activity in the brain was determined. After tail suspension test, the MAO activity was upregulated significantly; the MAO activity in the groups treated with tail suspension and then followed by moderate, high dosage decoction from* C. deserticola* Y.C.Ma showed a significant decline by approximately 13.9%~12.3%; the MAO activity in the groups treated with tail suspension and then high dosage decoction from* C. tubulosa* showed significant decline by about 13.2% (Figure 4). The data exhibited that decoction from both* C. deserticola* Y.C.Ma and* C. tubulosa *could inhibit the activity of MAO and may contribute to the antidepressant effect, and decoction of* C. deserticola* Y.C.Ma showed a stronger efficacy than that of* C. tubulosa*.

### 3.5. The Administration of* C. deserticola* Y.C.Ma and* C. tubulosa* Decoction Resulted in the Upregulation of Central Dopamine Concentration and the Downregulation of Serum Corticosterone Concentration

**Figure 5:**
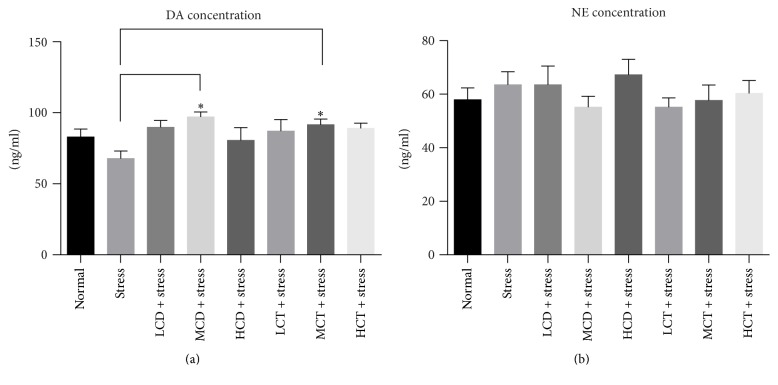
Effect of* C. deserticola* Y.C.Ma and* C. tubulosa* decoctions on brain neurotransmitter levels of tail suspension stressed mice. After adaptation, the experimental groups were administered with herb* Cistanche* decoction by gavage for 21 days and then suspended by tail for 5 min, each group was comprised of 10 mice, and the mice were sacrificed to harvest the brain tissues and used for brain neurotransmitter level measurement. L, M, and H, respectively, represent low, medium, and high; CD and CT, respectively, represent* C. deserticola* Y.C.Ma and* C. tubulosa* decoction. *n* = 10; all the data were represented as mean ± SEM; *P* < 0.05 was considered as significantly different and was marked as “*∗*”.

The level of DA and NE in the central nervous system was measured to determine the nerve excitability. After 4 weeks of tail suspension test, compared to the stress groups, the DA level of groups treated with moderate dosage* C. deserticola* Y.C.Ma was upregulated significantly each by 43.5%; the DA level of groups treated with moderate dosage* C. tubulosa *was upregulated significantly, too (Figure 5(a)). The level of NE was mildly upregulated in the groups treated with decoction of both herbs compared to the stress groups, and no statistical difference was found (Figure 5(b)). These results might explain the molecular mechanism of antidepressant effect of herb* Cistanche*.

**Figure 6:**
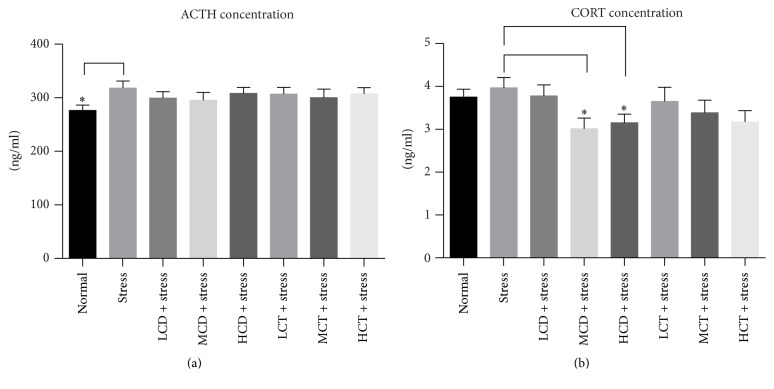
Effect of* C. deserticola* Y.C.Ma and* C. tubulosa* decoctions on serum hormone concentrations in hanging stressed mice. After adaptation, the experimental groups were administered with herb* Cistanche* decoction by gavage for 21 days and then suspended by tail for 5 min, each group was comprised of 10 mice, and the blood was harvested and used for determination of serum adrenocorticotrophic hormone (ACTH) and corticosterone (CORT) level. L, M, and H, respectively, represent low, medium, and high; CD and CT, respectively, represent* C. deserticola* Y.C.Ma and* C. tubulosa* decoction. *n* = 10; all the data were represented as mean ± SEM; *P* < 0.05 was considered as significantly different and was marked as “*∗*”.

Adrenocorticotropic hormone (ACTH), a polypeptide hormone produced and secreted by the anterior pituitary gland, stimulates secretion of glucocorticosteroid by acting on the adrenal cortex, zona fasciculata [31]; corticosterone, a steroid hormone produced by the cortex of the adrenal glands, is involved in the regulation of the physiological processes such as energy boosting, immunity reaction, and stress responses [32], so we chose to study the effect of herb decoction on the HPA axis. And ACTH and corticosterone concentration in the serum were measured. After 4 weeks of tail suspension test, the ACTH and corticosterone concentration of the stress group were significantly upregulated (Figure 6). No significant changes were observed in the herb* Cistanche* decoction treatment groups compared to the stress group (Figure 6(a)). Compared to the stress group, the corticosterone serum concentration of groups treated with moderate, high dosage* C. deserticola* Y.C.Ma decoction was downregulated significantly; the corticosterone concentration of groups treated with high dosage* C. tubulosa* was downregulated but not to a significantly statistical difference (Figure 5(b)). The data showed that decoction from both* C. deserticola* Y.C.Ma and* C. tubulosa *could reduce the corticosterone serum concentration and might be involved in regulating the HPA axis, and decoction of* C. deserticola* Y.C.Ma showed a stronger efficacy than that of* C. tubulosa*.

## 4. Discussion

In this study, the antidepressant activities and improvement of memory and learning of herb* Cistanche* were defined, which might shed a new light on herb* Cistanche* function and give clues to expanding the therapeutic application range. Previous studies show that herb* Cistanche* is a multifunctional Chinese drug with biological activities of antiaging, antioxidant, estrogenic, antiosteoporotic, aphrodisiac, and anti-inflammation effects [33]. Herb* Cistanche* has been recognized as a treatment of kidney deficiency, infertility, and chronic constipation (Pharmacopoeia Committee of China, 2010). Herb* Cistanche* decoction is composed of volatile oils, nonvolatile phenylethanoid glycosides (PeGs), iridoids, lignans, alditols, oligosaccharides, and polysaccharides. Echinacoside and verbascoside are generally viewed as the main bioactive ingredients [34]. There are four main* Cistanche* species including* C. deserticola *Y.C.Ma,* C. tubulosa*,* Cistanche sinensis* G. Beck, and* Cistanche salsa *var.* albiflora*shi et al. [35]; we chose* C. deserticola *Y.C.Ma and* C. tubulosa *as our model to determine the PeGs content, and* C. deserticola *Y.C.Ma possessed a higher content of PeGs (Figure 1), which may explain the higher efficacy of* C. deserticola* Y.C.Ma. The results indicate that the content of the main bioactive ingredients determines the efficacy of herb* Cistanche*.

Previous studies indicate that herb* Cistanche* products are beneficial for treating neurodegenerative disorders by upregulating nerve growth factor (NGF) in the hippocampus of mice [36, 37], but no research has been reported on the antidepressant and cognitive learning activities of herb* Cistanche* in the mammal models. In this study, herb* Cistanche* decoction was proven to be effective to alleviate the stress level and substantially increase the survival behavior in tail suspension test (Figure 2) and improve the spatial learning and memory capacity in the water maze model (Figure 3). Lin et al. proved that herb* Cistanche* improved the olfactory-associated memory in the fruit-fly model [23]; we focused our research on the antidepressant effect and adopted a different animal model; the outcome proved that herb* Cistanche* improved a variant type of cognitive function, namely, the spatial learning ability in mice. According to the monoamine theory, the stress experience and depression are closely linked to the brain level of monoamine neurotransmitters, such as DA, NE, 5-HT, and epinephrine [38]. And we found that the herb* Cistanche* decoction could undermine the MAO activity (Figure 4) and augment the central DA concentration (Figure 5(a)). Surprisingly, the central 5-HT concentration was downregulated by herb* Cistanche* decoction treatment (data not shown), because previous research showed that the reduced central 5-HT activity is associated with depression [39]. Dysregulation of the hypothalamic-pituitary-adrenal axis is frequently associated with depression [40], intervention with the glucocorticoid receptor (GR) system successfully reversed the depressant phenotype in mice [41], and we found that herb* Cistanche* significantly alleviated the serum concentration of CORT (Figure 6(b)). Modulation of the monoamine system and HPA axis both contributes to the antidepressant effect of herb* Cistanche*. The echinacoside could cross the blood-brain barrier freely and act upon the central nervous system [42], which may explain why herb* Cistanche* decoction possessed an antidepressant function. For the first time, we characterized the antidepressant property and its relationship to cognitive improvement activities in the mouse model, and the preliminary mechanism was explored. Still, the mechanism of antidepressant property of* Cistanche* has not been clarified on the molecular level, and further research is needed to elucidate the comprehensive antidepressant mechanism of herb* Cistanche*.

Considering that herb* Cistanche* decoction is mixture of compounds, there might be multiple ways of herb* Cistanche* decoction exerting the antidepressant effect and boosting learning ability. Oxidative stress affects many cellular functions of neurons, and overproduction of reactive oxygen species (ROS) causes cell damage, apoptosis, and death [43]. Echinacoside extracted from the stems of* C. salsa *exhibits a neuroprotective effect by reducing production of ROS and mitochondria-mediated apoptosis [44]; acteoside, an extremely strong antioxidative compound, might be partly responsible for the neuroprotective effect [45]. Besides the traditional method of decoction, application of modern pharmaceutic methods such as controlled release glycoside capsules might boost the effect of herb decoction, because the bioactive ingredients could be digested and degraded by the gastrointestinal tract [37].

## 5. Conclusions

In this study, we found that decoctions of* C. deserticola* Y.C.Ma and* C. tubulosa *exhibited an antidepressant effect and improved the spatial learning and memory ability in the mouse model, and decoctions of* C. deserticola* Y.C.Ma and* C. tubulosa *had a significant impact on the HPA axis;* C. deserticola *Y.C.Ma showed a stronger pharmacological function.* C. deserticola* and* C. tubulosa *might hold the potential of being developed as alternative therapy drug for depression.

## Figures and Tables

**Table 1:** The content of echinacoside and verbascoside in the powder and decoction of *C. deserticola* Y.C.Ma and *C. tubulosa*.

Samples	PeGs%	
	ECH%	VerBS%
*C. deserticola* powder	1.27 ± 0.009	0.52 ± 0.003
*C. tubulosa* powder	19.81 ± 0.66	2.45 ± 0.12
*C. deserticola* decoction	0.90 ± 0.001	0.20 ± 0.001
*C. tubulosa* decoction	5.90 ± 0.12	0.54 ± 0.05

Echinacoside, ECH; phenylethanoid glycosides, PeGs; verbascoside, VerBS.
